# Gallbladder motility and the sex of the guinea pig

**DOI:** 10.14814/phy2.12843

**Published:** 2016-06-28

**Authors:** Loren Kline, Edward Karpinski

**Affiliations:** ^1^ University of AlbertaSchool of DentistryEdmontonAlbertaCanada; ^2^ Department of PhysiologyUniversity of AlbertaEdmontonCanada

**Keywords:** Dihydrotestosterone, estrogen, gallbladder, progesterone, smooth muscle

## Abstract

Progesterone (P), 17*β*‐estradiol (E2), and dihydrotestosterone (DHT) affect gallbladder motility. When gallbladders were taken from women and men, women had more estrogen and P receptors than men. Both P and E2 had an inhibitory effect upon gallbladder contractility in men and premenopausal and postmenopausal women. Similar findings have been reported in gallbladder strips from male and female guinea pigs. In the present study, there was no significant difference in the amount of E2‐, P‐, or DHT‐induced relaxation of CCK‐induced tension when the responses in gallbladder strips from male and female guinea pigs were compared. Three metabolites of P were used: 17‐hydroxyprogesterone (17‐P), 20*α*‐hydroxyprogesterone (20‐P), and 21‐hydroxyprogesterone (21‐P). There was no significant difference in the responses from strips from male and female guinea pigs. In order to determine if the effects of E2 and P were additive, strips from male animals were exposed to either E2 or P and the amount of relaxation recorded. After recovery, the strips were exposed to E2 or P in reverse order to ensure the order of treatment had no effect. Then, the strips were treated with both E2 and P simultaneously and the relaxation recorded. This procedure was repeated with strips from female guinea pigs. The effect of E2 and P was found to be additive; however, the response of the strips from each sex were not significantly different. It is concluded that the sex of the guinea pig has no significant effect on the response to the sex hormones used.

## Introduction

Sex differences in normal physiological function have long been recognized. Such differences are well recognized in both the cardiovascular and pulmonary systems (Farhat et al. 1996; Townsend et al. 2012). Very little has been described concerning differences in the gastrointestinal system. Females are disproportionately affected by constipation, which is often aggravated during pregnancy. Bowel function also changes during the luteal phase of the menstrual cycle (Gonenne et al. 2006). The sex hormones 17*β*‐estradiol (E2), progesterone (P), and dihydrotestosterone (DHT) have been shown to affect the motility of the gallbladder. Gallstones are more common in women than men (Friedman et al. 1966). A decrease in the motility of the gallbladder has been observed during pregnancy. This decrease in motility was attributed to elevated P levels. (Mann and Higgins 1927; Gerdes and Boyden 1938; Kern et al. 1981). Age, gender, and female sex hormones are thought to influence contractility of the gallbladder. Estrogen receptors have been described in human gallbladder tissue (Singletary et al. 1986; Messa et al. 1990). Keane et al. (1986) observed that P and E2 had an inhibitory effect on gallbladder motility. The gallbladders used were taken from males, premenopausal females, and postmenopausal women due to gallstones or mild chronic cholecystitis. Ranelletti et al. (1991) found that gallbladders taken from women had more estrogen receptors than men. In addition, the gallbladders taken from women also had more receptors for P than men. Svoboda et al. (2008) showed that human gallbladder tissues contain enzymes and estrogen receptors *α* and *β* which together could regulate estrogen concentrations in human gallbladder.

Hyperandrogenemia has also been found to cause a decrease in gallbladder motility (Isik et al. 2012). Perusquia et al. (2005) suggested that testosterone (T) and DHT inhibited spontaneous contractile activity in pregnant human myometrium by inhibiting L‐type Ca^2+^ channels. Seyrek et al. 2007 demonstrated that T relaxed isolated human radial artery by opening potassium channels. T and DHT were also shown to inhibit the motility of male guinea pig gallbladder strips in vitro (Kline and Karpinski 2008). Both T and DHT inhibited CCK‐induced tension in a concentration‐dependent manner. Multiple pathways including inhibition of intracellular Ca^2+^ release, inhibition of Ca^2+^ entry, and the actions of PKC mediated the relaxation.

Kline and Karpinski (2011), using male guinea pig gallbladder strips, demonstrated that E2 relaxed CCK‐ or KCl‐induced tension in a concentration‐dependent manner. The inhibition of extracellular Ca^2+^ entry was shown to mediate the E2‐induced relaxation. Messa et al. (1990) demonstrated that estrogen reduced the maximal contractile response to CCK‐induced tension. Kline and Karpinski (2013) showed that DHT, P, E2, 17‐hydroxyprogesterone (17‐P), and 20*α*‐hydroxyprogesterone (20‐P) induced a concentration‐dependent relaxation of CCK‐induced tension. DHT, E2, and P also induced a concentration‐dependent relaxation of KCl‐induced tension. Inhibition of extracellular Ca^2+^ entry mediated the E2‐induced relaxation of both the CCK‐ and KCl‐induced tension. Inhibition of tyrosine kinase and PKA mediated the P‐induced relaxation in male guinea pig gallbladder strips. In the female strips inhibition of extracellular Ca^2+^ entry also had a role.

Thus, studies have suggested that the effects of E2 and/or P on gallbladder motility may have had a causal role in the increased incidence of gallstones in women. It has now been demonstrated that E2 directly affects hepatocyte function to enhance cholelithogenesis (Wang et al. 2004). However, the effects of E2 and P on motility may still have a contributing role in cholelithogenesis. The purpose of this study was to determine if there were differences in gallbladder motility in response to the sex steroids (E2, P, DHT, 17‐P, 20‐P, or 21‐hydroxyprogesterone [21‐P]) in male and female guinea pig gallbladder strips precontracted with CCK.

## Material and Methods

This research was approved by the Animal Care Committee, University of Alberta and awarded protocol #275 (renewed January, 2016). Young female (215–365 g body weight) and male (210–360 g body weight) Hartley guinea pigs were used in the study, and were killed by decapitation. The gallbladder was removed, fat and connective tissue removed, and placed in Krebs–Henseleit solution (KHS) that was gassed with 95% O_2_ and 5% CO_2_. The composition of the KHS was (mmol/L) NaCl, 115; KCl, 5; CaCl_2_, 2.1; MgSO_4_, 1.2; NaH_2_PO_4_, 1.2; NaHCO_3_, 25; and glucose, 11. Each gallbladder was cut into strips (1.5 × 0.5 cm) and maintained in Sawyer‐Bartlestone chambers filled with KHS, maintained at 37°C, and gassed with 95% O_2_ and 5% CO_2_. An optimum resting tension of 0.7 g was determined previously and used in the study (Kline and Karpinski 2005).

The force developed by the gallbladder strips was measured with Grass FT03 force displacement transducers and recorded on a Grass 7D polygraph (Grass Instruments Co., Quincy, MA). Isolated strips were equilibrated in the chambers for 45 min prior to determining their suitability for use. Each chamber had 2 *μ*mol/L (final concentration) atropine added, in every experiment, 3 min prior to either 1.0 nmol/L CCK or 40 mmol/L KCl (Kline et al. 1991). The tension was measured. This was followed by three changes in KHS. The test was repeated twice with 25 min between tests. A repeatable minimum tension of 0.5 g had to be generated by the strips if they were to be used. All agents used were added directly to the chambers. All concentrations are reported as the final concentration in the chambers.

Several series of experiments were performed to determine the amount of relaxation of CCK‐induced tension caused by E2, P, 17‐P, 20‐P, 21‐P (17‐P, 20‐P, and 21‐P are all metabolites of P), and DHT. CCK (1 nmol/L) was found to produce a stable long‐lasting tension after 3 min. This steady tension lasted at least 10 min; that is, there was no measurable decrease in the observed tension during the 10 min (Kline et al. 1991). Concentration–response curves were generated in a manner similar to previously reported (Kline and Karpinski 2005, 2008, 2011, 2013) for each hormone used. The CCK‐induced tension was allowed to reach a steady level (3 min). The strips were exposed to 1 concentration of DHT, P, 17‐P, 20‐P, 21‐P, or E2, the response was observed until the relaxation reached a steady level (approximately 5 min), the KHS was changed three times, and the strips were allowed to recover for 30 min before testing a different concentration of DHT, P, 17‐P, 20‐P, 21‐P, or E2.

In order to determine if the effects of E2 and P on gallbladder motility were mediated through different signaling pathways, male guinea pig gallbladder strips were exposed to E2 (50 *μ*mol/L) and the amount of relaxation of 1.0 nmol/L CCK‐induced tension was recorded. After a 25‐min recovery period, the strips were then administered E2 (50 *μ*mol/L) and P (50 *μ*mol/L) as close to the same time as possible and the amount of relaxation of CCK‐induced tension was recorded. The procedure was repeated using P (50 *μ*mol/L) initially and then the combination of E2 and P. This procedure determined that the order of E2 or P exposure had no effect on the observed responses.

All agents were purchased from Sigma‐Aldrich (St. Louis, MO): CCK, atropine, E2, P, 17‐P, 20‐P, 21‐P, E2, and DHT. All agents were dissolved in either distilled water or DMSO. The amount of DMSO (10 *μ*L) added to the chambers was determined to have no effect on the strips.

Results are expressed as a mean ± the standard error of the mean (SEM). Statistical comparisons were done using either the *t*‐test or analysis of variance (SigmaPlot v. 13, Systat Software, Inc., San Jose, CA). The number of gallbladders (animals) used in each experiment are indicated by “*n*.” Each gallbladder was used to prepare four strips. Differences among mean values with *P *<* *0.05 were considered significant.

## Results

**Figure 1:**
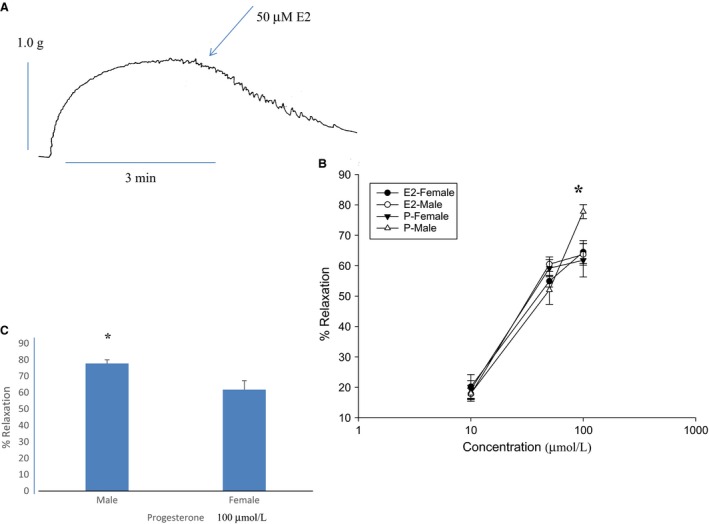
The effect of E2 or progesterone (P) on CCK‐induced tension. (A) A data trace showing the relaxation caused by E2 on CCK‐induced tension in a gallbladder strip from a male guinea pig. (B) E2 relaxed CCK‐induced tension in strips taken from male (open circles) and female (filled circles) guinea pigs. There was no significant difference (*n* = 11) in the amount of E2‐induced relaxation when the responses of strips from male and female guinea pigs were compared at any concentration used. P relaxed CCK‐induced tension in gallbladder strips taken from either male or female guinea pigs. When 10 or 50 *μ*mol/L P was used there was no significant difference in the amount of P‐induced relaxation when the responses of the strips from male and female guinea pigs were compared. (C) When 100 *μ*mol/L P was used strips taken from male guinea pigs relaxed significantly (*P* < 0.001, *n* = 13) more than strips taken from female guinea pigs.

E2 caused a relaxation of CCK‐induced tension (Fig. 1A and B). This E2‐induced relaxation was concentration dependent in gallbladder strips taken from either male or female guinea pigs. There was no significant difference, when responses from each sex were compared, in the amount of E2‐induced relaxation at any concentration (10, 50, or 100 *μ*mol/L, *n* = 11) of E2 used.

P induced a concentration‐dependent relaxation of CCK‐induced tension in strips from either male or female guinea pig gallbladder strips (Fig. 1B). When either 10 *μ*mol/L or 50 *μ*mol/L P was used there was no significant difference between the male‐ and female‐derived strips. However, when 100 *μ*mol/L P was used, the strips taken from male guinea pig gallbladders were relaxed significantly (*P* < 0.01, *n* = 17) more than those strips taken from female guinea pigs (Fig. 1C). The response of the strips taken from male guinea pigs to 100 *μ*mol/L P was also significantly (*P* < 0.01) more than that to 100 *μ*mol/L E2 in either male‐ or female‐derived strips (Fig. 1B).

**Figure 2:**
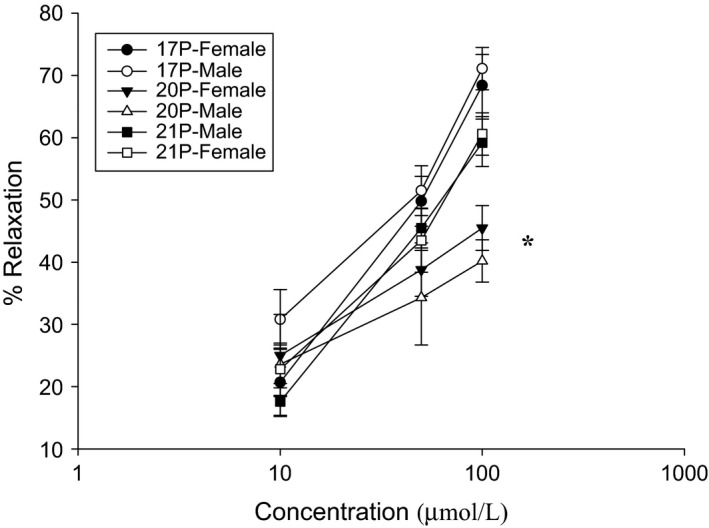
The effects of three metabolites of P on CCK‐induced tension. At all concentrations (10, 50, and 100 *μ*mol/L) of 17‐P (*n* = 9), 20‐P (*n* = 8) and 21‐P (*n* = 10) used, there was no significant difference in the amount of relaxation of CCK‐induced tension when the responses of strips taken from male and female guinea pigs. The responses of strips taken from both sexes to 20‐P were significantly (*P* < 0.001) less than the responses to either 17‐P or 21‐P.

Several metabolites of P were used. The responses to 17‐P (*n* = 9), 20‐P (*n* = 8), and 21‐P (*n* = 10) can be seen in Figure 2. The strips taken from both male and female guinea pigs responded to each P metabolite in a concentration‐dependent manner. When the responses of the strips taken from male guinea pigs were compared with those taken from female guinea pigs for each metabolite were compared, there was no significant difference in the responses to each concentration used. The responses to 100 *μ*mol/L 20‐P by strips from both male and female guinea pigs were significantly (*P* < 0.01) less than the responses from strips taken from male and female guinea pigs to 17‐P and 21‐P.

**Figure 3:**
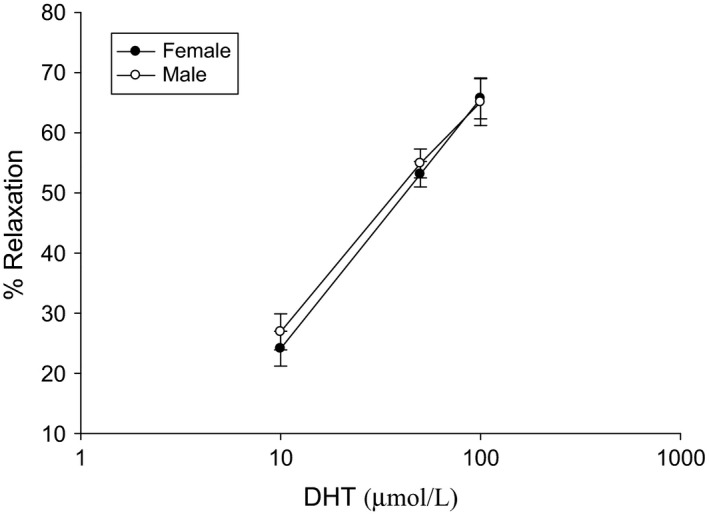
The effect of dihydrotestosterone (DHT) on CCK‐induced tension. DHT relaxed CCK‐induced tension in gallbladder strips from both male and female guinea pigs. There was no significant difference at any concentration (10, 50, and 100 *μ*mol/L, *n* = 20) when the responses from each sex were compared.

DHT relaxed CCK‐induced tension in a concentration‐dependent manner in strips taken from both male and female guinea pig gallbladders. There was no significant difference in the amount of DHT‐induced relaxation at any concentration used (10, 50, and 100 *μ*mol/L, *n* = 20; Fig. 3) when the responses from strips from each sex were compared at each of the concentrations used.

In order to determine if the effects of E2 and P were additive, strips taken from male guinea pigs were exposed to either 50 *μ*mol/L E2 or 50 *μ*mol/L P, and the amount of relaxation of 1.0 nmol/L CCK‐induced tension recorded. After the recovery period those strips exposed to 50 *μ*mol/L E2 were then treated with 50 *μ*mol/L P and those initially treated with 50 *μ*mol/L P were exposed to 50 *μ*mol/L E2. This was to ensure that the order of treatment with either E2 or P had no effect. After the recovery period, the strips were then treated with both 50 *μ*mol/L E2 and 50 *μ*mol/L P together and the relaxation recorded. The results can be seen in Table 1. There was no significant difference observed when the amount of E2‐induced relaxation was compared with the amount of P‐induced relaxation. When the amount of E2‐induced relaxation of CCK‐induced tension was compared with that from strips exposed to both E2 and P in combination, there was significantly (*P* < 0.001, *n* = 14) more relaxation observed. Likewise, when the amount P‐induced relaxation was compared with that obtained when both E2 and P were used in combination, there was significantly (*P* < 0.001) more relaxation than when P was used alone (Table 1).

**Table 1:** The effect of E2 or P on the relaxation of male guinea pig gallbladder strips (E2 and P were used separately and in combination)

	E2	P	E2 + P
% Relaxation	47.9 + 2.6	47.4 + 3.0	66.3 + 1.9
E2 vs. E2 + P	*P* < 0.001		
P vs. E2 + P	*P* < 0.001		
E2 vs. P	NS		

The percentage of relaxation induced by E2 or P are shown as is the amount of relaxation induced by using both E2 and P together. The amount of relaxation when both agents were used together was compared with the relaxation when E2 or P was used alone. There was significantly more relaxation observed than with individual agent. The order of the treatment with either E2 or P had no role.

## Discussion

The concentrations of E2, P, P metabolites, and DHT used in this study may not be considered physiologic; however, it has been argued that the short‐term application of high concentrations of steroids/factors can mimic low (physiologic) levels applied over a long period of time (Kline and Karpinski 2005).

Women have a higher incidence of gallstones than men (Gerdes and Boyden 1938). Pregnancy decreases the in vivo contractile activity of the human gallbladder (Mann and Higgins 1927; Gerdes and Boyden 1938; Kern et al. 1981). This was attributed to elevated P levels. The guinea pig gallbladder contains P receptors that are responsive to physiological concentrations of P and have a regulatory effect on gallbladder contractility (Hould et al. 1988). Davis and Ryan (1986) showed that P significantly decreased the maximal contractile response to both ACh and CCK, but had no effect on the ED_50_ of either concentration–response curves. It was suggested that P modulates extracellular Ca^2+^ entry. This finding was supported by Kline and Karpinski (2013) using strips taken from female guinea pig gallbladders. Other second messenger systems were also found to be involved (Kline and Karpinski 2005). It has now been shown that the effects of P are of a similar magnitude in strips taken from both male and female guinea pigs, with exception that when 100 *μ*mol/L P was used the strips taken from male guinea pig was significantly (*P* < 0.001 more than the response to the same concentration of P by strips from female guinea pigs. The response to 100 *μ*mol/L P in the male guinea pig gallbladder strips may have been due to the interaction of the high concentration of P with T receptors (McGuire et al. 1977). The amount of relaxation of CCK‐induced tension caused by 17‐P, 20‐P, or 21‐P was not significantly different when the responses from strips take from each sex were compared.

T and DHT have nongenomic actions on smooth muscle. In human myometrial smooth muscle T, 5*α*‐DHT, and 5*β*‐DHT caused a concentration‐dependent inhibition of spontaneous contractile activity. These androgens relaxed KCl‐induced tension. The inhibition of L‐type Ca^2+^ channels mediated the effect (Perusquia et al. 2005). In rat middle cerebral arteries, T modulated the vascular tone in these arteries using an endothelium‐derived hyperpolarizing factor (Gonzales et al. 2004). T and DHT potentiated the contractile activity in mouse ileal smooth muscle strips via nongenomic actions. The potentiating mechanism was mediated through polyamine signaling secondary to stimulation of ornithine decarboxylation and Ca^2+^ sensitization via Rho kinase activation (Gonzales‐Montelongo et al. 2006). Testosterone has been shown to relax porcine coronary arteries predominantly by opening large‐conductance, calcium‐activated K^+^ channels (BK_Ca_). The response may be associated with cGMP accumulation (Farhat et al. 1996). In the human radial artery, testosterone relaxed KCl‐induced tension. This relaxation was mediated in part by ATP‐sensitive K^+^ channel opening (Deenadayalu et al. 2001). DHT was shown to relax CCK‐ and KCl‐induced tension in a concentration‐dependent manner in gallbladder strips taken from both male and female guinea pigs (Kline and Karpinski 2005, 2011). In the present study, there was no significant difference in the amount of relaxation DHT induced when the responses from each sex were compared.

Estrogen is present in both sexes (Wibowo and Wasserug 2014). Estrogen therapy, including the use of oral contraception and postmenopausal estrogen therapy, has been shown to be a risk factor for gallbladder disease (Cirillo et al. 2005). Observational studies indicated up to a 2.5‐fold increased risk of biliary tract conditions related to estrogen therapy (Kakar et al. 1988; Grodstein et al. 1994; Nelson et al. 2002). Estradiol inhibits voltage‐dependent Ca^2+^, Ca^2+^‐dependent K^+^, and voltage‐dependent K^+^ currents in pregnant rat myometrium (Okabe et al. 1999). Estrogen also decreased contractility in human colonic smooth muscle (Hogan et al. 2009). E2 interfered, in a concentration‐dependent manner, with Ca^2+^ contractile effects in male aortic rings stimulated with *α*_1_‐adrenergic and serotonergic receptors. E2 inhibited the capacitative Ca^2+^‐influx through both L‐type Ca^2+^ and non‐L‐type Ca^2+^ channels (Castillo et al. 2006). Kline and Karpinski (2005, 2013) demonstrated that E2 relaxed CCK‐ and KCl‐induced tension in a concentration‐dependent manner. Gallbladder strips taken from both sexes of guinea pig responded to E2 in a manner such that there was no significant difference in the amount of relaxation observed and any concentration used. Pietras and Szego (1977) demonstrated that E2 receptors were present on the membrane of isolated endometrial cells. Wang et al. (2004) demonstrated that estrogen enhanced cholesterol cholelithogenesis by augmenting functions of hepatic estrogen receptor *α* on the hepatocyte membrane.

In coronary arteries from female rhesus monkeys, P produced a rapid vasodilator action (Minshall et al. 2002). P produced a relaxation in both male and female rat aortic strips. The effect was considered nongenomic (Selles et al. 2002). Previous studies demonstrated that the P‐induced relaxation of CCK‐induced tension in strips taken from male guinea pig gallbladders was mediated through tyrosine kinase and the PKA/cAMP second messenger pathway (Kline et al. 1991). In addition, E2 relaxed CCK‐ and KCl‐induced tension in gallbladder strips taken from male guinea pig gallbladders. This relaxation was mediated by E2 inhibiting Ca^2+^ entry (Kline and Karpinski 2013). Estradiol inhibited voltage‐dependent Ca^2+^ currents in pregnant rat myometrium (Okabe et al. 1999). Salom et al. (2002) found that E2 relaxed the rabbit carotid artery. The relaxation was mediated by inhibition of extracellular Ca^2+^ influx.

The presence of the E2 G‐protein‐coupled estrogen receptor (GPER) has been implicated in the decreased incidence of many diseases in premenopausal women (Prossnitz and Barton 2011). The hepatic estrogen receptor ER*α*, but not ER*β*, was shown to have a critical role in E2‐induced cholesterol gallstones (Wang et al. 2004). The use of estrogen therapy in postmenopausal women led to an increased risk of gallstones. The authors suggested that the morbidity and cost associated with these outcomes should be considered in decisions regarding estrogen therapy (Cirillo et al. 2005). The results of the studies by Prossnitz et al., Cirillo et al., and others (Wang et al. 2009) suggested that the levels of E2 may have a greater role in gallstone formation in women than changes in gallbladder motility.

In the present study, by using E2 and P together it has been shown that the effects of the two hormones are additive, suggesting that each is exerting its effect by different pathways.

## Conclusions

It has been shown that there is little difference in the response of male and female guinea pig gallbladder strips to P, 17‐P, 20‐P, 21‐P, DHT, or E2. When the amount of relaxation induced by each hormone was compared for each concentration used there was no significant difference with only one exception, that is, the response of strips taken from male guinea pigs to 100 *μ*mol/L P. The response to this concentration of P was significantly greater than that of strips taken from female guinea pigs. Since there was little, if any, difference in the responses of gallbladder strips taken from female guinea pigs when compared to those from male guinea pigs, it is concluded that there is no difference in motility; and thus, motility may have little effect on the incidence of gallstones.

## Conflict of Interest

The authors have no conflicts of interest.
